# Phytomedicines and conventional drugs in scabies management

**DOI:** 10.3205/dgkh000625

**Published:** 2026-02-17

**Authors:** Majedul Hoque

**Affiliations:** 1 Department of Pharmacy, Jahangirnagar University, Dhaka, Bangladesh

**Keywords:** scabies, treatment, natural products, Pongamia pinnata, essential oils

## Abstract

**Background and aims:**

In some countries, the prevalence of scabies varies from 32.1% to 74%, especially in overcrowded institutions like boarding schools, prisons, and orphanages. The aim of this article is to give an overview of current options for scabies treatment, with the focus on natural treatment choices.

**Method:**

Different research databases including Google Scholar, PubMed, Springer, Science Direct, and NIH also scientific website were searched using keywords like “scabies”, “natural treatment for scabies”, “plant-based oil”, “chemical agent for scabies”, “essential oil used to treat scabies mite”, and “traditional medicine” to collect relevant information.

**Results:**

A lot of medicinal plants have been reviewed for their potential use in treating scabies, including *Melaleuca alternifolia*, *Curcuma longa*, *Azadirachta indica*, *Rosmarinus officinalis*, *Capsicum annuum*, *Ocimum sanctum*, *Pongamia pinnata*, and *Citrus limon*. Numerous bioactive chemicals found in all of the plants under study have the potential to treat scabies and can be used to treat this illness.

**Conclusion:**

Medicinal herbs offer a potential, safer, and possibly more successful therapeutic alternative for the treatment of scabies along with conventional chemical drugs because of their richness of bioactive components with antibacterial qualities. This area of research must be continued.

## Zusammenfassung

**Hintergrund:**

In einigen Ländern liegt die Prävalenz der Scabies (Krätze) zwischen 32,1% und 74%, insbesondere in überbelegten Einrichtungen wie Internaten, Gefängnissen und Waisenhäusern. Im Artikel soll ein Überblick über die aktuellen Möglichkeiten zur Behandlung der Scabies gegeben werden, wobei der Schwerpunkt auf natürlichen Behandlungsmethoden liegt.

**Methode:**

Verschiedene Forschungsdatenbanken, darunter Google Scholar, PubMed, Springer, Science Direct und NIH, sowie wissenschaftliche Webseiten wurden mit Stichwörtern wie „Scabies“, „natürliche Behandlung von Scabies“, „pflanzliches Öl“, „chemisches Mittel gegen Scabies“, „ätherische Öle zur Behandlung von Scabiesmilben“ und „traditionelle Medizin“ durchsucht.

**Ergebnisse:**

Viele Heilpflanzen wurden hinsichtlich ihrer potenziellen Verwendung zur Behandlung von Scabies untersucht, darunter Melaleuca alternifolia, Curcuma longa, Azadirachta indica, Rosmarinus officinalis, Capsicum annuum, Ocimum sanctum, Pongamia pinnata und Citrus limon. Zahlreiche bioaktive Chemikalien, die in allen untersuchten Pflanzen gefunden wurden, haben das Potenzial zur Behandlung der Scabies und können zur Behandlung dieser Krankheit eingesetzt werden.

**Schlussfolgerung:**

Heilpflanzen bieten aufgrund ihres Reichtums an bioaktiven Inhaltsstoffen mit antibakteriellen Eigenschaften eine potenzielle, sicherere und möglicherweise erfolgreichere therapeutische Alternative zur Behandlung der Scabies als herkömmliche chemisch synthetisierte Arzneimittel. Dieser Forschungsbereich muss daher weiterverfolgt werden.

## Introduction

Scabies is one of the most neglected diseases in the world [1]. More than 200 million people worldwide are thought to have contracted scabies at some point in their lives. Scabies can affect anywhere between 0.2 and 71% of people. According to a 2015 Global Burden of Disease Study, Indonesia has the highest rate of scabies infection out of all 195 nations worldwide [2]. Worldwide, new cases of scabies are more common in underdeveloped nations. 

This highly contagious ectoparasitic skin disease is caused by *Sarcoptes scabiei* var. hominis. *Sarcoptes scabiei* var. canis can occasionally adjust to humans, causing scabies infestations in those with weakened immune systems. 

There are still just a few therapy choices accessible. Benzyl benzoate, sulfur compounds, lindane (γ-benzene hexachloride), monosulfiram (tetraethyl thiuram monosulfide), crotamiton (crotonyl-N-ethyl-o-toluidine), lindane (an organophosphate insecticide), and permethrin (a synthetic pyrethroid insecticide) are examples of frequently used scabicides [3], [4]. 

Topical treatments are the mainstay of conventional treatment, while oral drugs may also be recommended in some circumstances to completely remove the mites. Permethrin cream is one of the most widely used topical scabicides [5]. Oral drugs like ivermectin may be taken into consideration when topical therapies are ineffective or impracticable [3]. Different nations have different medication availability, which causes variations in treatment methods [6]. Conventional therapies might have negative consequences, such as skin irritation and itching, even when they are effective [5]. The impact of mass treatment programs and the therapeutic relevance of resistance to antiscabies medications like permethrin and ivermectin are still being studied and debated. Resistance to these medicines is becoming a more serious problem [6]. 

Scabies therapy is still challenging, particularly in tropical and subtropical regions [7]. Topical medications, benzyl benzoate 10–25% or permethrin 5% cream, or oral ivermectin are the mainstays of contemporary human scabies therapies [8]. Nonetheless, there are more and more reports available about parasite resistance to several important scabicides, such ivermectin and pyrethroids. Therefore, it is imperative to create alternate strategies for scabies control [9], [10], [11]. As a consequence, alternative approaches to conventional acaricides are needed and essential oils from plant and plant based products have been considered among other compounds. Plants’ antibacterial, anti-inflammatory, and antioxidant qualities make them highly promising for the management and treatment of burns and wounds [12]. Medicinal herbs provide a safe, cost effective, and patient-friendly natural therapy alternative for scabies since they are rich in bioactive components [13]. The purpose of this article is to give an overview of scabies treatment, with a unique focus on natural treatment choices for better scabies prevention initiatives.

## Method

The literature search was conducted across multiple scientific databases, including Google Scholar, PubMed, Springer, ScienceDirect, and the National Institutes of Health (NIH), covering publications up to June 2025. The search strategy employed a combination of relevant keywords such as “scabies”, “natural treatment for scabies”, “plant-based oil”, “essential oil used to treat scabies mite”, “chemical agent for scabies” and “traditional medicine”. In addition to peer-reviewed journal articles, pertinent books and scientific literature were also reviewed to ensure a broad coverage of available evidence. The collected data were systematically analyzed, interpreted, and critically discussed to synthesize the current understanding and highlight emerging insights regarding the natural and synthetic treatment approaches for scabies. 

## Result and discussion

### Epidemiology and risk factors for scabies

Worldwide, scabies is a common condition that is believed to impact over 200 million individuals simultaneously, with around 455 million new cases occurring annually [14]. In developed nations, scabies occurrences are recorded but infrequent, and it is typically not regarded as a major public health concern. Within the general populace, the prevalence of scabies in European and Middle Eastern countries is reported to be low (<2%), and it is undiscovered if this is attributed to socioeconomic factors or climatic conditions [15]. Recent data, however, have indicated that the frequency of scabies has increased in certain nations, particularly among those with weakened immune systems or in settings where outbreaks are more frequent, such as hospitals, nursing homes, schools, and jails. The number of scabies cases in Germany has significantly increased over the last 20 years, with an estimated 200% rise in treated outpatient cases reported in the year 2014 and 2016 [16]. According to a national survey, there are between 80,000 and 150,000 scabies cases in Japan each year [17]. The nationwide frequency is thought <1%, despite the fact that institutional outbreaks are still common [18]. In addition to impeding the procedures of diagnosis, case management, contact tracing, and epidemic identification, the overcrowded living conditions and frequent transfers in facilities like jails allow illnesses, particularly scabies infestations, to spread [19]. Similar findings have been reported among vulnerable groups including refugees and asylum seekers as well as in European refugee camps, where scabies has been identified as one of the most common dermatological presentations in Germany, the Netherlands, and Greece [20], [21], [22], [23]. In Germany, scabies as an acquired occupational disease among medical personnel rose from no cases in 2002 to 172 in 2017 [24].

Many low- and middle-income nations have endemic scabies, especially those with tropical climates where poverty and cramped living conditions are prevalent and access to effective treatment is restricted [25]. According to a nationwide study conducted in Fiji, 43.6% of children aged 5 to 9 had a scabies diagnosis, whereas the incidence in the overall population was 23.6% [26]. According to a study conducted in Bangladesh, patients experienced social isolation and feelings of embarrassment as a result of the stigma and shame surrounding scabies, which had a moderate impact on their quality of life. Compared to children, all of these abnormalities were more commonly seen in adult patients [27]. Prior to the intervention, the prevalence of scabies in Bangladeshi madrasahs was 61% and 62%, but following widespread scabies treatment, the prevalence dropped to 5% and 50%. The intervention at madrasahs also significantly improved personal hygiene behaviors [28]. 

There is no evidence suggesting that scabies is notably more prevalent in either males or females. Some research has identified minor differences, likely stemming from variations in circumstances, exposure risks, and behaviors. In certain environments, males may exhibit higher rates of prevalence due to greater exposure in communal living situations such as military barracks, prisons, or specific occupational settings, while in other contexts, females may face a higher risk of contracting scabies. Given their roles as primary caregivers in households, women may have more frequent and prolonged contact with children who are often carriers of scabies, placing them at a greater risk of infestation in such vulnerable situations. The likelihood of infection rises in areas with high population density, including nursing homes, schools, prisons, refugee camps, and communities experiencing overcrowded living conditions. In these environments, re-infestation through contact with untreated individuals or family members is common [29]. Individuals who are immunosuppressed or immunodeficient have a higher likelihood of developing crusted scabies, which is the rarer and more severe variant of the condition [30]. Long-term direct skin-to-skin contact is the primary way that scabies is spread, however sharing clothes, towels, or beds can also occasionally be a factor [31]. Successful transmission occurs after about 20 min of intimate contact (such as holding hands, breastfeeding a baby, sharing a bed, or having intercourse), and it spreads readily across homes, schools, and medical facilities [32], [33]. 

### Issues related to the use of conventional scabicides

Women who are pregnant as well as children under the age of 15 years should not use ivermectin [34]. P-glycoprotein inhibitors including methotrexate, cyclosporine, digoxin, and some anticancer medications can be extremely harmful when used with ivermectin [35]. Compared to permethrin, crotamiton 10% cream is less effective [36]. Crotamiton’s safety for usage in neonates and babies is not fully proven. Also, following a single treatment for eight to twelve hours, substantial resistance rates have been seen [37]. A single topical application of lindane has been reported to have a cure rate of 49% to 96%; nevertheless, resistance is mostly to blame for treatment failures [38]. The medication lindane has major neurotoxic side effects such vertigo, convulsions, agitation, vomiting, diarrhea, and syncope [39]. In vitro, benzoyl benzoate, a synthetic chemical, exhibits more miticidal action than permethrin [40]. Because it is a highly irritating substance that can result in contact dermatitis, it should be diluted before being applied to children or women who are pregnant or nursing, and it should be removed within 24 hours [41]. 

### Anti-scabies mode of action

The inhibition of cytochrome P450 monooxygenases and reduction of ecdysone hormone production are the causes of the insecticidal and acaricidal action. One of the main hormones involved in insect molting is ecdysone (20-hydroxyecdysone), which when suppressed resists an insect from growing further and ultimately results in its death. By breaking down the poisons that cause mites to become resistant to different pesticides, cytochrome P450 monooxygenases aid in detoxification. Certain medications block cytochrome P450 monooxygenase, which raises the amount of dangerous medicine in insects and eventually results in their demise [42]. Permethrin produces sustained depolarization of nerve cell membranes by interfering with voltage-gated sodium channels, which disrupts nerve impulses and results in paralysis. Crotamiton is believed to reduce itching by causing a counter-irritation effect when it evaporates off the skin, giving the skin a cooling effect, however the precise process is not entirely known. Additionally, it could inhibit the itching pathways triggered by histamine and chloroquine [43], [44]. Certain drugs must stay on the skin for a certain amount of time in order to kill newly formed larvae, which hatch from eggs a few days after treatment.

### Common medicinal plants and potential phytochemicals for the treatment of scabies 

More than 3.3 billion people use medicinal plants daily in less developed nations since they are extremely useful to our lives and the foundation of traditional medicine [45]. 80% of people worldwide use natural or herbal remedies for their medical needs [46]. Compared to allopathic medications, natural or herbal treatments are less expensive and are said to be safe with few or no adverse effects. Active ingredients include glycosides, alkaloids, flavonoids, coumarin compounds, vitamins, and tannins give medicinal plants their therapeutic potential [47]. 

Many species were utilized as herbal remedies in traditional medical systems, either as an extraction or as the entire plant [48]. These therapeutic herbs are being used by locals and specialists with traditional knowledge. Conventional information provides useful insights for scientific research. Traditional knowledge about the medical applications of plants is extremely helpful to understand the pharmacological importance of medicinal herbs. Nowadays people are using different medicinal plants to treat scabies in several ways (Table 1 (Tab. 1)).

Some of the medicinal plants with scabicidal activities contain prominent antimicrobial biochemical compound that might be effective against *Sarcoptes scabiei* described in the (Table 2 (Tab. 2)). 

### Herbal plant based essential oil effective against scabies

Essential oils are the secondary metabolic byproducts that are stored in various sections of plant and act as protection against certain diseases. For pesticidal methods, almost 17,500 aromatic plant species may be significant [49]. These oils, which are mostly composed of a blend of various terpenes, sesquiterpenes, and aromatic chemicals including phenols and phenylpropanes, give plants their distinctive scent and may be extracted using solvents, distillation, and mechanical squeezing [50]. These substances have a variety of biological effects, some of which are noteworthy include antibacterial, antiviral, antifungal, and antiparasitic effects, which refer to a direct action against pathogens, as well as advantageous effects on patients (such as antioxidant, anti-inflammatory, and immunomodulant effects), which indirectly aid in healing [51]. 

In vitro and in vivo studies have examined a number of essential oils from various medicinal plants in various animal species to check against *S. scabiei*. Table 3 (Tab. 3) provides a summary of the most relevant findings for scabicide activities.

### Conventional pharmaceutical agent for the treatment of scabies

The two main synthetic medication therapies for scabies are topical permethrin cream or oral ivermectin. They used as first line therapy. 5% permethrin cream is safe for adults, pregnant women, and children older than two months. It is applied to the entire body from the neck down. Another alternative is oral ivermectin, particularly for crusted scabies or in cases where topical treatments are ineffective. However, its safety for pregnant women and children <15 kg has not been proved [52], [53]. Some chemically synthesized compound that demonstrated acaricidal activity against S. scabiei are summarized in Table 4 (Tab. 4). 

Since mites appear to avoid locations with a high density of sebaceous glands and significant seborrhea, topical treatments should be given to the entire body, excluding the face and scalp. At least 8 to 12 hours must pass while the therapy is applied to the skin [54]. The acaricidal effects of a 1% aqueous creolin emulsion containing 2.5–3% gamma benzene hexachloride and a 2% aqueous solution of trichlorofon were tested in captive-bred Arctic foxes.

Two times at intervals of seven to eight days, animals with scabies infestations were immersed in the treatment solution for two to three minutes. The fur of the animals was completely cured with no negative consequences [55]. Usually, lindane lotion is only used when all other options for treating scabies have been exhausted. During pregnancy or nursing its usage is not advised. Another topical treatment is crotamiton cream or lotion, which often has to be used twice, separated by 24 hours. A topical remedy for scabies is sulfur ointment. It is usually regarded as safe for usage in both pregnant women and newborns. Usually containing 5% to 10% sulfur, sulfur ointments are applied all over the body and kept on for a predetermined amount of time, usually overnight, before being rinsed off.

### Potential nanoherbal formulation for scabies treatment

Potential nanoherbal formulations for scabies combine plant-based extracts with nanotechnology, primarily in the form of nanoparticles or nanoemulsions, to improve medication efficacy, stability, and transport while lowering adverse effects. Numerous herbs have shown potential in ongoing research. Among these formulations, nanoemulsions and SLNs are the most advanced, while green-synthesized nanoparticles are still in early experimental phases (Table 5 (Tab. 5)). 

### Preventive measures for scabies

Although scabies is not considered a fatal condition, it significantly impacts the patient’s quality of life; therefore, efforts for elimination and prevention are crucial. One method to eradicate scabies is by enhancing community understanding and awareness regarding the disease and the preventive steps, such as the proper handling of contaminated items (bedding, clothing, towels). To ensure the removal of mites, contaminated items should be laundered in hot water and dried using a hot dryer. If hot water is unavailable, killing can be achieved by sealing infected items in a plastic bag for seven days, since mites can only survive for three days outside of a host. Given that scabies are often found in boarding schools, involving non-medical staff in scabies screening could be beneficial for the early identification of cases. The use of a screening checklist for the signs and symptoms of scabies may be effective for early detection, thus facilitating prompt treatment [56]. 

Raising awareness through campaigns is crucial to preventing and controlling scabies outbreaks, especially in crowded areas like refugee camps and religious boarding schools. Good personal hygiene practices, such as frequent hand washing and avoiding sharing personal items, are crucial in halting the spread of scabies (Table 6 (Tab. 6)). People need to be made aware of the symptoms of scabies and how close skin contact can transmit the disease. Scabies can be prevented from spreading by having access to clean water, soap, and hygienic facilities. Intimate physical contact can be reduced by creating distinct spaces for individuals and families. Distribution of medications, such as topical permethrin, can help keep outbreaks going, but it should be used in conjunction with measures to deal with unsanitary conditions.

### Future directions

**Figure 1:**
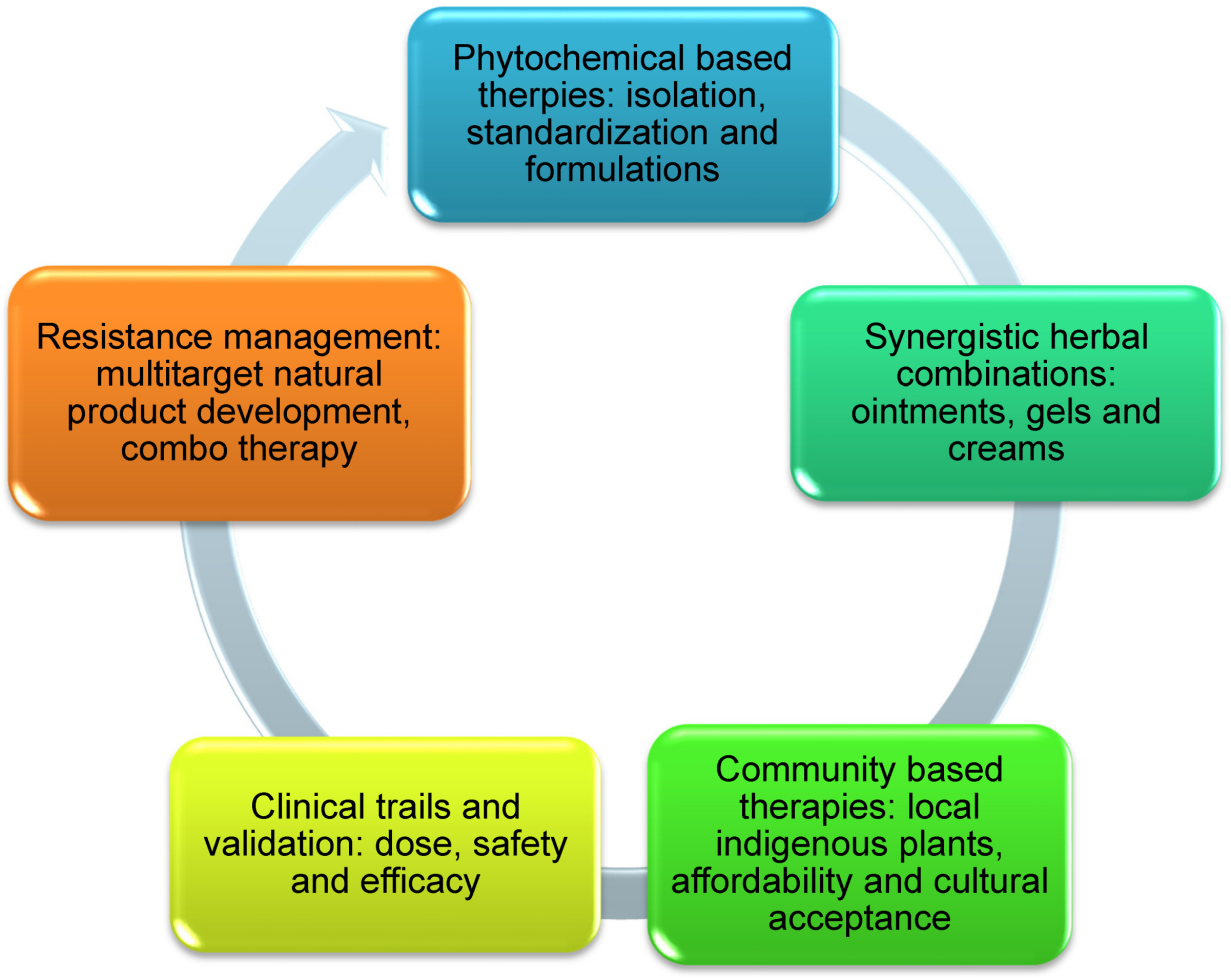
Diagram for scabies management by natural means

The future of treating scabies with natural therapies hinges on community integration, safe formulation, regulatory approval, and scientific assurance (Figure 1 (Fig. 1)). While natural products offer promising alternatives to conventional drugs, overcoming challenges of standardization, clinical evidence, toxicity, and global recognition is essential for their successful adoption. 

## Conclusion

Both natural and synthetic treatments can be used to treat scabies. Even though pharmaceutical therapies like permethrin and ivermectin are commonly prescribed and have been shown to be helpful, natural remedies including tea tree oil, neem oil, lemon oil, and clove oil have also shown promise. The decision between them frequently comes down to personal preferences, the extent of the infestation, and the profile of adverse effects. Although natural medication has less efficacy and time consuming regimen but they possess lower adverse effects compared to allopathic medicine, so natural products might be a viable source for the treatment of scabies. The review discussed various plant based agents, phytochemicals and synthetic formulations that have been used for the treatment scabies in various species of mammals on the basis of reports from different researches and literatures. 

From a green standpoint, essential oils are easy solutions that are biodegradable, have low ecotoxicity, and have little environmental residual activity because of their high volatility. Finally it can be concluded that, a promising, safer, and maybe more successful therapy option are provided by medicinal plants, which are abundant in bioactive chemicals with antimicrobial qualities. More researches are required to be continued in this aspect of science. 

## Notes

### Author’s ORCID

- Hoque M: https://orcid.org/0009-0001-9044-411X

### Funding: 

None. 

### Competing interests

The author declares that he has no competing interests.

## Figures and Tables

**Table 1:**
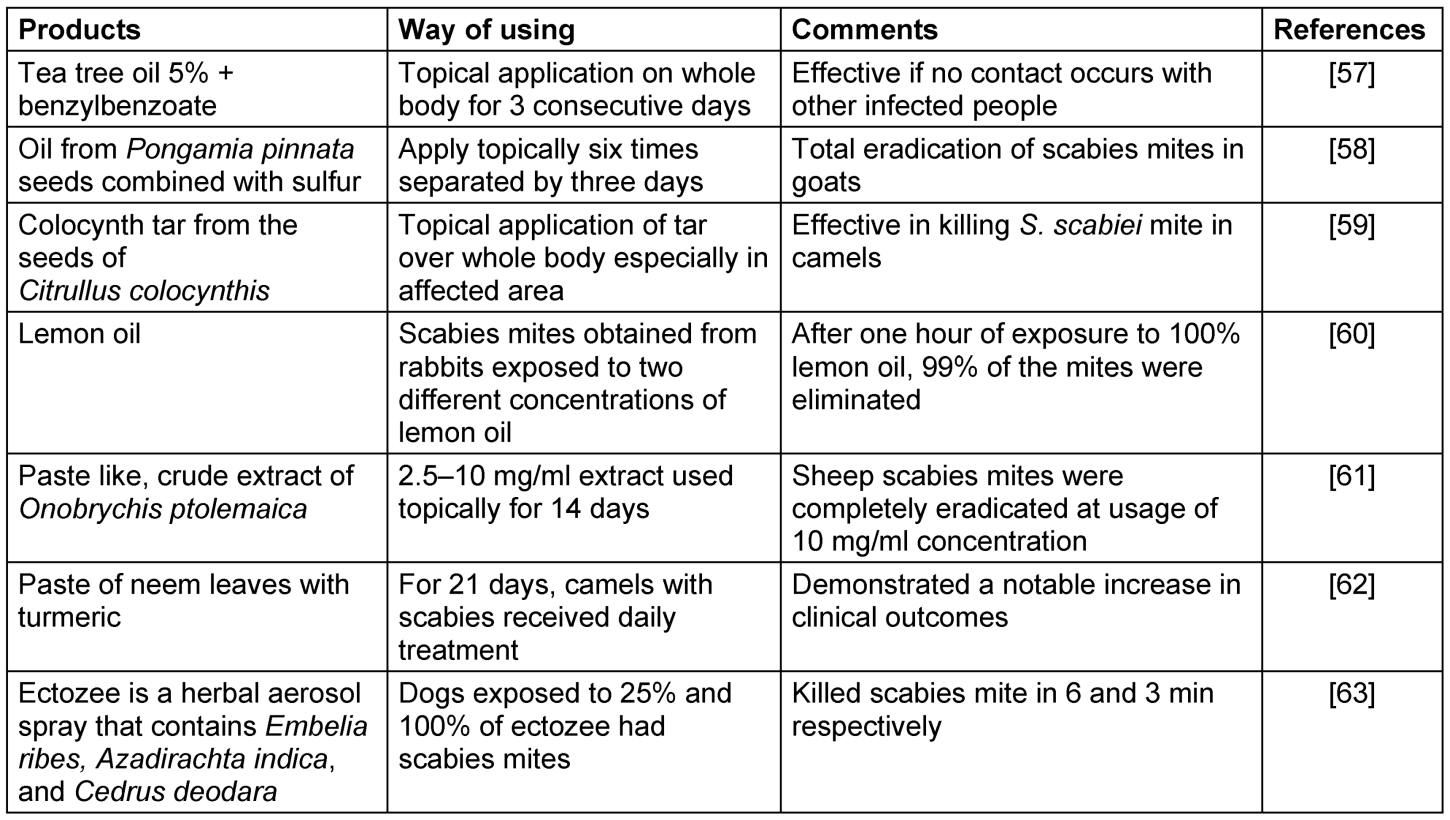
Natural plant based medicinal product demonstrated as effective for the treatment of scabies

**Table 2:**
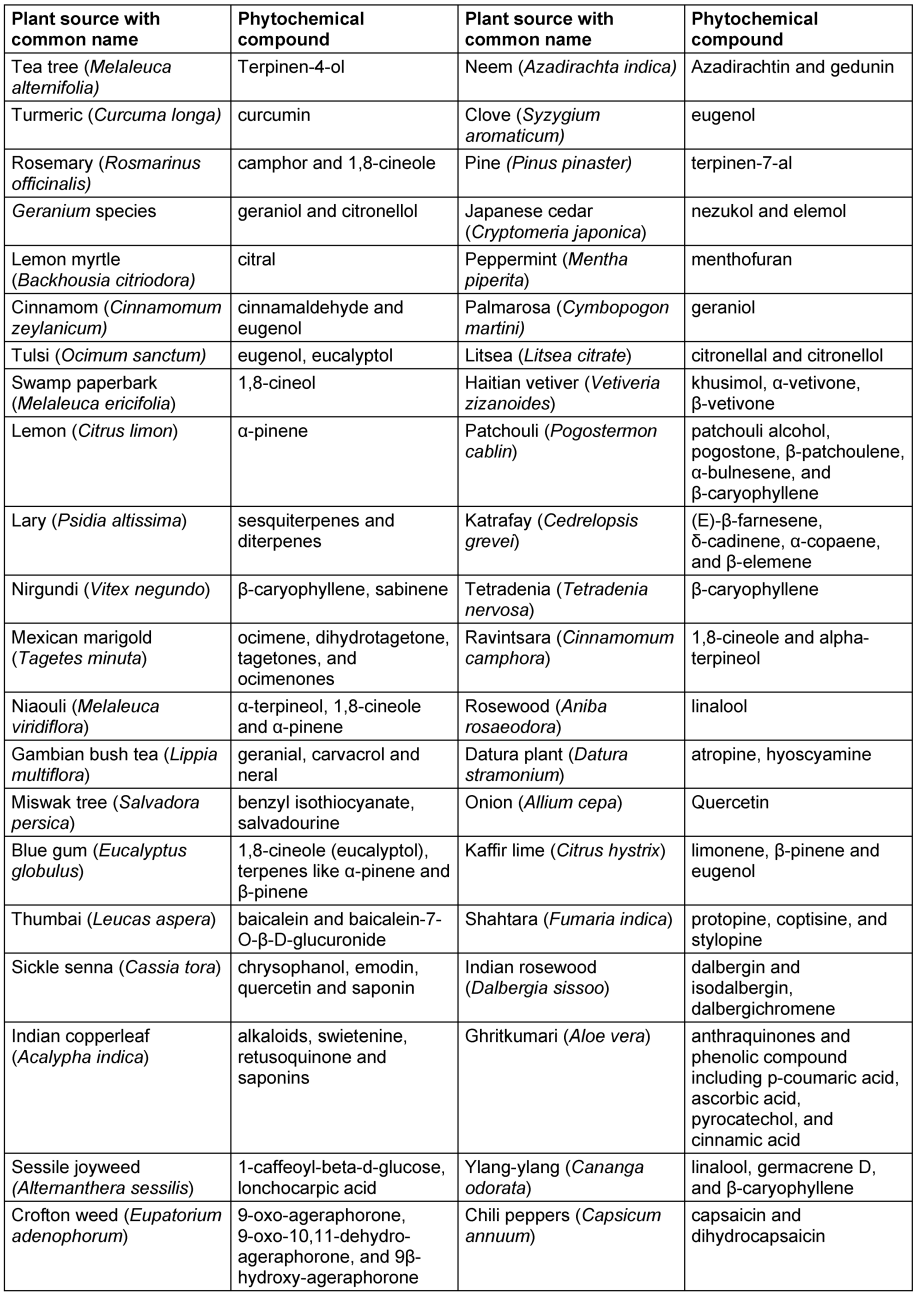
Prominent antibacterial phytochemical compound found in medicinal plants act against scabies mite

**Table 3:**
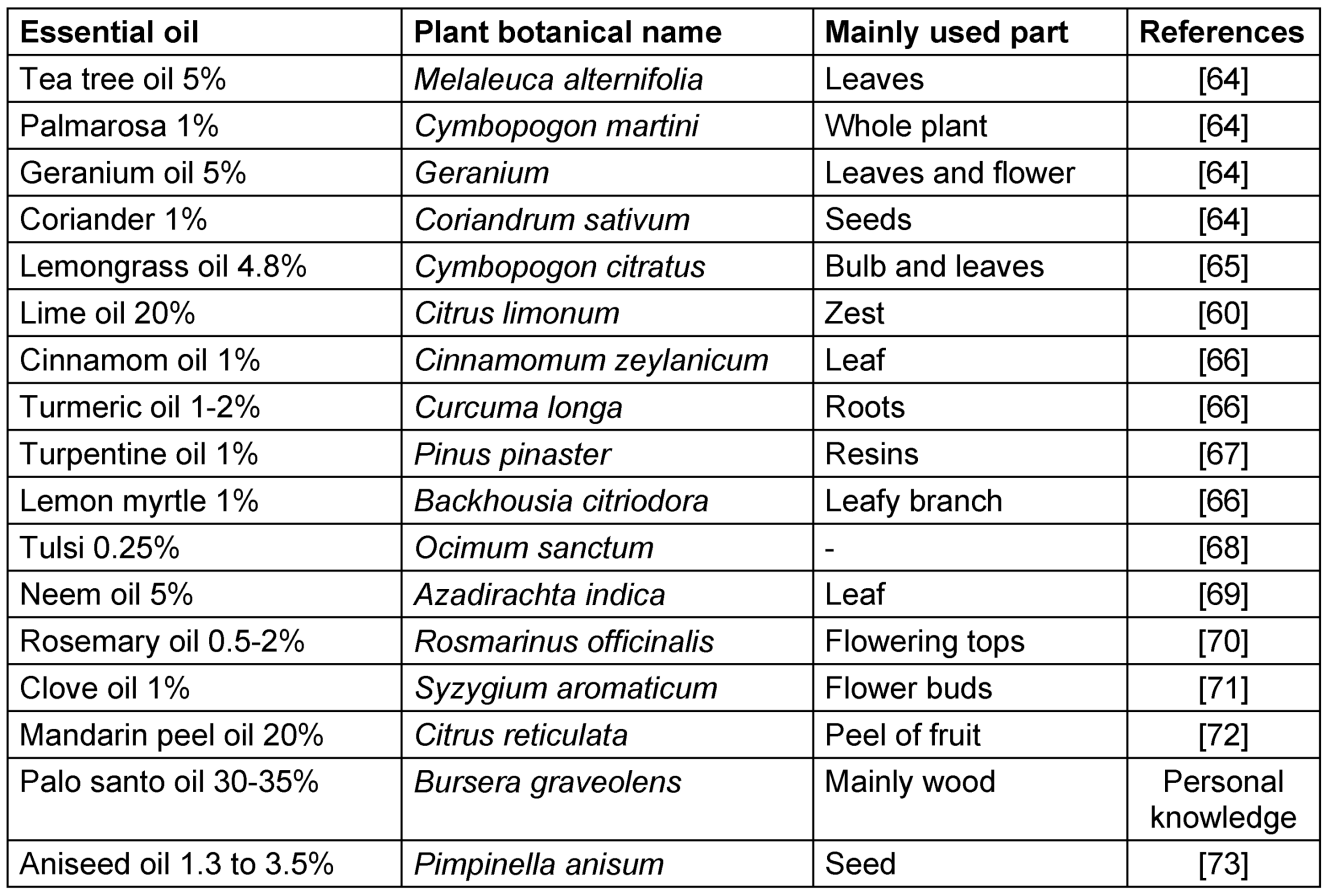
Plant based essential oil effective against scabies

**Table 4:**
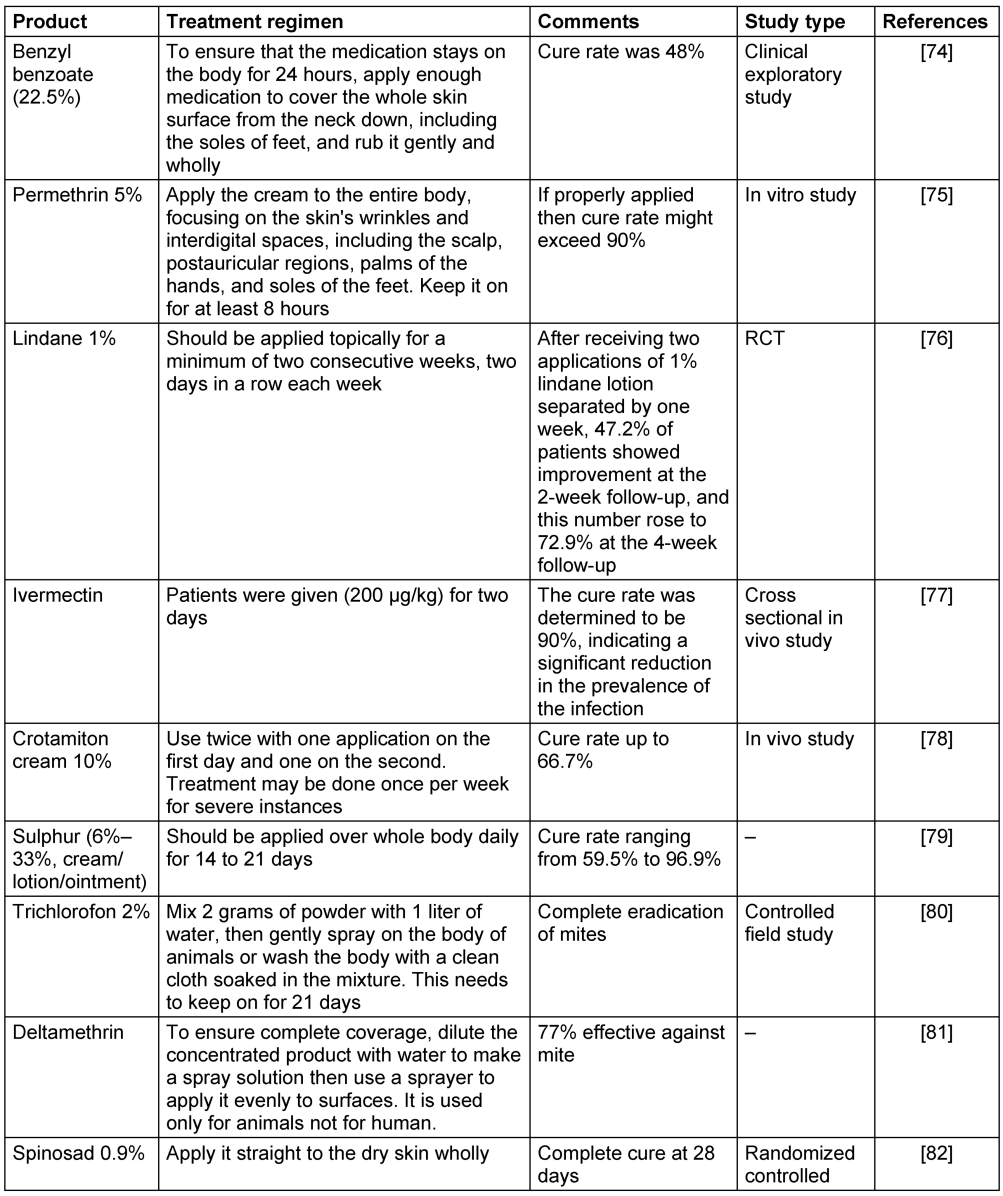
Most commonly used effective synthetic agent used to treat scabies infection

**Table 5:**
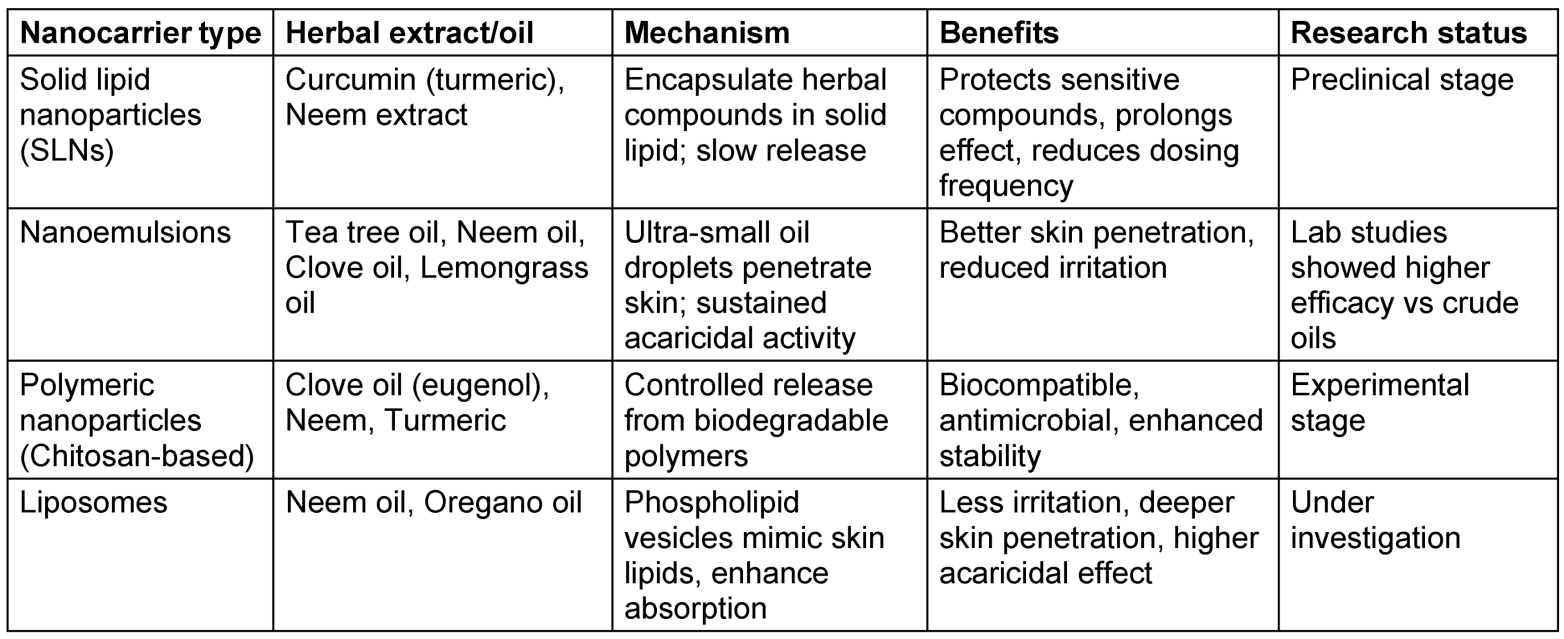
Nanotechnology-based herbal formulations for scabies treatment

**Table 6:**
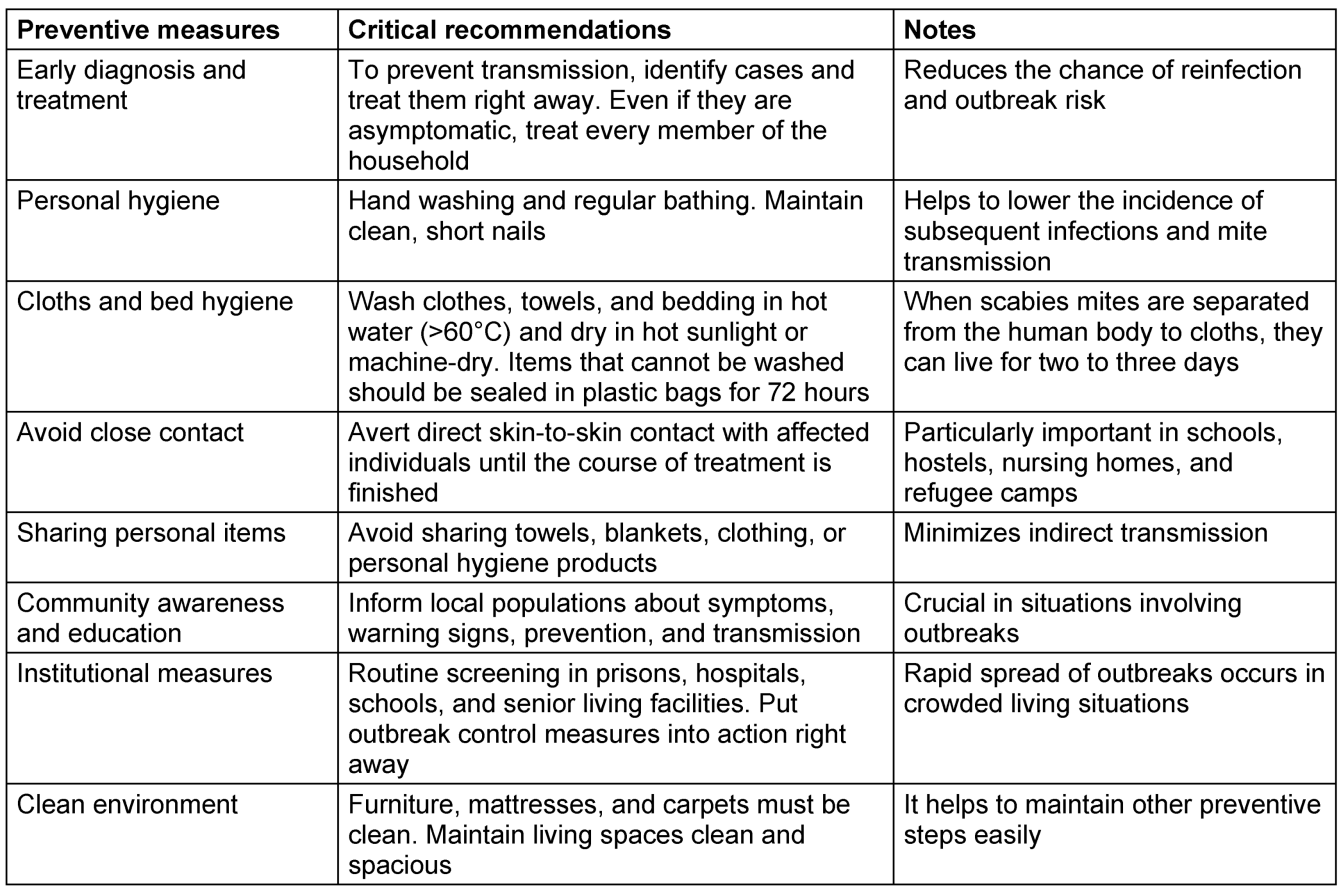
Few steps and critical recommendations for scabies prevention
